# The Impact of Stone Composition on Treatment Strategies for Patients with Urolithiasis: A Narrative Review

**DOI:** 10.7759/cureus.75345

**Published:** 2024-12-08

**Authors:** Marius Ivanuta, Dragos Puia, Octavia Petrila, Ana-Maria Ivanuta, Catalin Pricop

**Affiliations:** 1 Urology, Grigore T. Popa University of Medicine and Pharmacy, Iasi, ROU; 2 Urology, "Dr. C.I.Parhon" Clinical Hospital, Iasi, ROU; 3 Center for Morphological and Spectroscopic Analysis of Urinary Stones “Michel Daudon”, "Dr. C.I.Parhon" Clinical Hospital, Iasi, ROU; 4 Radiology, "Dr. C.I.Parhon" Clinical Hospital, Iasi, ROU; 5 Emergency Medicine, "St. Spiridon" County Emergency Clinical Hospital, Iasi, ROU

**Keywords:** alkaline citrate, prevention of kidney stones, shock wave lithotripsy, stone free, urinary stone analysis

## Abstract

Urolithiasis is a multifactorial condition where stone composition is critical in guiding treatment and prevention strategies. Advanced diagnostic techniques, such as infrared spectroscopy, provide precise stone analysis, enabling clinicians to tailor interventions based on specific stone types and associated metabolic abnormalities. Calcium oxalate monohydrate stones often require invasive approaches like percutaneous nephrolithotomy, while uric acid responds well to dissolution therapy. Cystine stones, linked to genetic cystinuria, necessitate lifelong management, including aggressive hydration, urinary alkalinization, and pharmacological treatments such as tiopronin. Stone composition significantly influences treatment decisions and recurrence prevention in urolithiasis. Integrating detailed compositional analysis into clinical practice enables personalized interventions, improving outcomes and reducing recurrence rates. Future research should focus on refining diagnostic techniques and exploring novel therapies to address the metabolic and environmental contributors to stone formation. Adopting a multidisciplinary, patient-centered approach is essential for advancing the management of urolithiasis.

## Introduction and background

Urolithiasis, or urinary stone disease, is a complex, multifactorial aetiology. It arises from various factors, including geographical location, lifestyle habits, socioeconomic status, anatomical abnormalities, and underlying medical conditions. These factors significantly influence the composition of urinary stones, impacting the clinical approach to treatment and prevention. Understanding these determinants is essential for effectively managing urolithiasis [1].

Given the importance of stone composition in guiding treatment and prevention strategies, it is strongly recommended that all patients with urolithiasis, especially those with a high risk of recurrence, undergo at least one detailed stone analysis. This approach allows clinicians to classify patients and devise evidence-based preventive measures accurately. The guidelines of significant urological associations support this recommendation. Both the European Association of Urology (EAU) and the American Urological Association (AUA) underscore the significance of stone composition analysis, highlighting its role in improving patient outcomes and reducing the burden of recurrence [2,3,4].

Modern techniques, such as infrared spectroscopy (FTIR) and morphological analysis, have emerged as methods for urinary stone evaluation, offering superior accuracy compared to older biochemical methods. Infrared spectroscopy precisely identifies crystalline phases, including the differentiation of calcium oxalate monohydrate from dihydrate or various forms of calcium phosphate, which biochemical methods often fail to achieve [3].

Moreover, FTIR is less prone to significant errors associated with chemical methods, which can misidentify rare stone types, such as drug-induced calculi or purine stones, and are unable to quantify the proportions of components in mixed stones [5]. An accurate analysis must assess the composition of all parts of the stone, including the core, inner layers, peripheral layers, and surface. Each region can provide valuable insights into the pathogenesis of stone formation. For example, the core composition may reveal the initiating factor of stone growth, while the peripheral layers reflect changes over time influenced by metabolic or environmental factors [6].

Urinary tract stones are primarily classified by their composition. The most common types are calcium stones, struvite stones (magnesium ammonium phosphate), uric acid stones, and mixed stones, with different mineral compositions in varying percentages [3]. Less frequently encountered stone types include those associated with specific conditions, such as indinavir-induced stones and brushite, cystine, and protein matrix stones. Each type of stone composition carries distinct implications for pathogenesis, treatment, and prevention, making accurate classification essential in clinical practice [7]. Stone analysis is a critical step in the management of urolithiasis. It requires both qualitative and quantitative evaluation of the stone's crystalline phases, their distribution within the stone, and its structural characteristics-a process referred to as morpho-constitutional analysis [8]. This detailed approach involves examining the stone's surface and cross-sectional morphology, enabling clinicians to classify the stone into specific morphological types that often correlate with underlying metabolic disorders or systemic diseases [9].

This narrative review aims to explore the implications of urinary stone composition on the selection and optimization of treatment strategies in patients with urolithiasis. It emphasizes how accurate compositional analysis informs individualized treatment approaches, mainly through advanced techniques such as infrared spectroscopy and morphoconstitutional analysis. By examining the relationship between stone composition, underlying metabolic conditions, and therapeutic interventions, the study seeks to underscore the role of precise diagnostics in guiding medical, surgical, and preventive strategies to improve clinical outcomes and reduce recurrence rates in urolithiasis management.

## Review

Calcium oxalate monohydrate 

Calcium oxalate monohydrate (COM) is one of the most identified crystalline forms in urolithiasis. COM stones are notably dense and hard, making them more resistant to extracorporeal shock wave lithotripsy (ESWL) fragmentation than other stone types, such as calcium oxalate dihydrate or uric acid stones. This inherent resistance necessitates careful consideration when selecting treatment modalities, often requiring alternative approaches like laser lithotripsy or percutaneous nephrolithotomy (PCNL) for adequate stone clearance, particularly in large or complex calculi cases [3]. One of the primary etiological factors of hyperoxaluria contributing to the formation of these types of kidney stones is primary hyperoxaluria (PH).

Hyperoxaluria represents a pathophysiological process in developing recurrent nephrolithiasis, nephrocalcinosis, and eventual progression to end-stage kidney disease (ESKD). PH is an autosomal recessive metabolic disorder characterized by defects in key enzymes responsible for oxalate metabolism. Genetic mutations in *AGXT *are the most prevalent cause, leading to PH type 1, while *GRHPR* deficiencies underlie PH type 2, and *HOGA1 *mutations are associated with PH type 3. These enzymatic deficiencies result in excessive systemic oxalate production and subsequent deposition in renal and extrarenal tissues, including bones, cardiovascular structures, and retina, causing significant organ dysfunction and clinical morbidity [10].

Elevated urinary oxalate levels, often resulting from dietary sources (such as rhubarb, dark chocolate, beetroot and spinach, which contain high amounts of oxalate) or gastrointestinal conditions (Crohn’s disease, intestinal dysbiosis, such as reducing Oxalobater Formigenes) or malabsorption syndromes (bariatric surgery), significantly increase the risk of COM stone formation [6]. Hypocitraturia, another critical factor, reduces the natural inhibition of calcium oxalate crystallization, further predisposing individuals to stone development. In such cases, treatment focuses on acute stone removal and addressing these underlying metabolic derangements to prevent recurrence [9]. Effective management of COM stones includes medical, dietary, and interventional strategies. Dietary modifications are a cornerstone of prevention, emphasizing the reduction of oxalate-rich foods such as spinach, nuts, and chocolate, alongside maintaining adequate calcium intake to bind oxalate in the gastrointestinal tract and reduce urinary excretion [11].

Pharmacological interventions often include potassium citrate supplementation, which enhances urinary citrate levels and inhibits calcium oxalate crystallization. In cases of recurrent stones or severe hyperoxaluria, specific treatments such as pyridoxine (vitamin B6) may lower oxalate production in individuals with primary hyperoxaluria. Citrate inhibits stone formation by forming soluble calcium citrate complexes, reducing calcium oxalate and calcium phosphate crystallization and restoring the inhibitory properties of urinary proteins like Tamn-Horsfall protein [12]. An essential aspect of hyperoxaluria management involves distinguishing isolated hyperoxaluria from cases accompanied by hypercalciuria, as their underlying pathophysiology and treatment approaches differ. In isolated hyperoxaluria, oxalate excretion occurs without concurrent calcium abnormalities, necessitating targeted interventions. One potential therapeutic strategy is the administration of oral calcium supplements, which bind intestinal oxalate to form insoluble calcium oxalate complexes, thereby reducing oxalate absorption and lowering urinary oxalate levels. This approach highlights the role of dietary and pharmacological modifications in managing oxalate burden and mitigating the risk of nephrolithiasis in affected individuals [13].

Follow-up genetic testing plays a pivotal role in managing patients with significant hyperoxaluria, particularly in confirming a diagnosis of PH type 1. PH type 1 is caused by mutations in the AGXT gene, which encodes the liver enzyme alanine-glyoxylate aminotransferase (AGT). Deficiency or dysfunction of AGT leads to the accumulation of glyoxylate, which is subsequently converted to oxalate, resulting in systemic hyperoxaluria and its associated complications. Genetic testing enables the precise identification of the underlying mutation, distinguishing PH type 1 from other types of hyperoxaluria or metabolic disorders. This distinction is crucial, as the treatment paradigm for PH type 1 has advanced significantly with the advent of RNA interference (RNAi) therapy. RNAi therapies like lumasiran are designed to reduce gene expression in oxalate overproduction. Specifically, lumasiran targets the messenger RNA (mRNA) of glycolate oxidase (GO), encoded by the HAO1 gene, thereby reducing the production of glyoxylate and its subsequent conversion to oxalate. This innovative approach directly addresses the metabolic imbalance at its source, significantly lowering oxalate levels in the liver and, consequently, in the urine.

In addition to RNAi therapy, genetic confirmation of PH type 1 facilitates a more personalized approach to patient management, including dietary modifications, optimization of hydration, and surveillance for systemic oxalate deposition. Furthermore, identifying PH type 1 early through genetic testing allows for timely consideration of advanced treatments, including liver transplantation, where RNAi therapy or conservative management fails to prevent disease progression [14,15].

Clinical studies demonstrate the efficacy of alkaline citrates, particularly potassium citrate, in reducing stone recurrence, promoting stone dissolution, and preventing the growth of residual fragments after ESWL or PCNL. In patients with residual stone fragments smaller than 4 mm, citrate therapy reduces the risk of stone growth and recurrence. Randomized controlled trials have shown that potassium citrate reduces stone formation rates by up to 100% in some studies and sustains a significantly higher stone-free rate than placebo over long-term follow-ups. For instance, with citrate treatment, the stone formation rate in idiopathic hypocitraturic calcium nephrolithiasis patients decreased from 1.2 to 0.1 stones per year. Furthermore, potassium citrate therapy after PCNL has been linked to reduced procedural costs due to decreased recurrence rates, with minimal gastrointestinal side effects such as mild diarrhoea or nausea. Patients treated with potassium-sodium citrate had significantly improved stone-free status and stabilised stone size during a 12-month follow-up period compared to untreated patients [16].

Hydroxycitric acid (HCA), a derivative of citric acid, is naturally present in tropical rainforest plants such as Garcinia cambogia and Hibiscus sabdariffa, which are valued for their edible and medicinal properties. HCA has been extensively studied for its potential to lower blood pressure and lipid levels, inhibit calcium oxalate crystal formation, and reduce renal damage. These findings suggest that HCA may serve as an effective preparatory treatment to increase stone fragility before ESWL, thereby improving treatment outcomes [17,18].

Calcium oxalate dihydrate stones 

Calcium oxalate dihydrate stones (COD) are one of the crystalline forms of calcium oxalate found in urinary stones alongside COM. Unlike COM, COD crystals are less dense and exhibit a more fragile structure, which makes them more amenable to fragmentation through ESWL. Despite their lower density, COD stones remain a significant clinical concern due to their propensity for aggregation and growth under specific metabolic conditions [3]. The formation of COD stones is primarily associated with hyperoxaluria, hypercalciuria, and other metabolic abnormalities, such as hypocitraturia. These conditions promote the supersaturation of calcium oxalate in the urine, leading to crystal nucleation, growth, and aggregation. COD crystals are also highly dependent on urinary pH and other environmental factors within the urinary tract, making their prevention and treatment closely tied to metabolic regulation [6]. The formation and stability of COD stones are closely linked to urinary pH, which plays a critical role in the crystallization and transformation of calcium oxalate phases. COD crystals are favoured in environments with a neutral to slightly alkaline pH (typically around 6), as these conditions enhance the solubility of calcium oxalate and promote the formation of the less stable dihydrate form [19,20].

However, as the urinary pH decreases, the likelihood of converting COD to the more stable and less soluble COM increases, leading to a higher risk of forming denser and harder stones more resistant to fragmentation [21]. From a clinical perspective, COD stones often represent an earlier stage in stone formation, as they can transition into the more stable COM form under certain conditions. Metabolic conditions that influence urinary pH are critical in forming COD stones. Hypocitraturia, commonly linked to a lower urinary pH, reduces the availability of citrate, a natural inhibitor of calcium oxalate crystallization. Citrate binds calcium to form soluble complexes, and its deficiency shifts the balance toward forming calcium oxalate crystals. Similarly, hypercalciuria and hyperoxaluria further promote stone formation, mainly when urinary pH is neutral. At this pH, oxalate solubility decreases, leading to supersaturation and nucleation of COD crystals, thereby increasing the risk of stone development. These interactions highlight the importance of managing urinary pH and metabolic abnormalities to prevent COD stone formation. Therapies aimed at maintaining a slightly alkaline urinary pH (6.5-7.2) using alkaline citrates, such as potassium citrate, have been shown to effectively reduce the risk of calcium oxalate crystallization by increasing calcium oxalate solubility and preventing supersaturation [6,7].

Thiazide diuretics, such as hydrochlorothiazide, chlorthalidone, and indapamide, reduce urinary calcium excretion by enhancing calcium reabsorption in the distal convoluted tubules of the kidney, thereby lowering the risk of calcium oxalate supersaturation and crystal formation. They may also increase urinary citrate levels by correcting metabolic acidosis, further inhibiting stone formation. Thiazides are particularly effective in managing idiopathic hypercalciuria, significantly reducing stone recurrence rates. However, long-term use may cause side effects like hypokalaemia, metabolic alkalosis, and hyperglycemia, which can be mitigated by combining thiazides with potassium supplementation or potassium-sparing diuretics [22,23]. Dietary factors also play a significant role in urinary pH regulation. An excessive animal protein intake lowers urinary pH, increasing the risk of stone formation, while a diet rich in fruits and vegetables buffers urinary pH and reduces the likelihood of calcium oxalate dihydrate stone development [24].

Uric acid stones 

Uric acid stones (UA) stones result from a combination of metabolic and dietary factors, with a persistently low urinary pH being the primary driver. When the urinary pH drops below 5.5, UA is predominantly insoluble, promoting crystal nucleation and stone formation. Hyperuricosuria, caused by high purine intake (organ meats such as liver, kidneys, certain seafood like anchovies, sardines, mackerel, mussels, scallops, trout, and herring) or metabolic disorders like gout, exacerbates this process by increasing the concentration of UA in urine. Insulin resistance, often seen in metabolic syndrome, impairs renal ammonia genesis, reducing the kidney's ability to buffer urine and contributing to acidic urinary conditions. These factors highlight the multifaceted nature of UA stone formation, requiring targeted interventions. The management of UA stones is centered on alkalinization therapy to increase urinary pH, as the solubility of UA improves significantly at a pH above 6.5. Potassium citrate is the treatment of choice for its alkalinizing effects and its ability to inhibit calcium oxalate crystallization, often associated with mixed stones. For patients with hyperuricosuria or recurrent stones, pharmacological options such as allopurinol reduce UA production by inhibiting xanthine oxidase [3,6,7].

Allopurinol and febuxostat are xanthine oxidase inhibitors widely used to manage hyperuricemia, including gout and uric acid nephrolithiasis. Allopurinol functions by inhibiting xanthine oxidase, the key enzyme responsible for converting hypoxanthine to xanthine and, subsequently, xanthine to uric acid. This inhibition reduces uric acid production, decreasing serum and urinary uric acid levels. Similarly, febuxostat acts as a selective xanthine oxidase inhibitor but demonstrates a higher affinity for the enzyme than allopurinol, providing potent and sustained suppression of uric acid synthesis. Both agents effectively lower uric acid levels, reducing the risk of gout flares and uric acid stone formation. Their pharmacological action targets the overproduction of uric acid and contributes to the dissolution of existing uric acid crystals by maintaining uric acid levels below the solubility threshold [25].

Studies have shown that patients with diabetes are at a higher risk of developing UA stones compared to non-diabetic individuals. This risk is exacerbated by common comorbidities, such as obesity and metabolic syndrome, which not only lower urinary pH but also increase uric acid production. Also, it indicates distinct differences in the formation and recurrence of uric acid stones between patients with type 1 diabetes and type 2 diabetes. Patients with type 1 diabetes predominantly form pure UA stones. In contrast, type 2 diabetes patients are more likely to develop mixed UA stones, a difference that is statistically significant and impacts treatment strategies, particularly for stone dissolution [26,27].

The frequency of uric acid stones is notably higher in the geriatric population, with UA stones accounting for 83% of cases in this group. This increased prevalence is primarily attributed to conditions such as metabolic syndrome and kidney impairment, which are more common in older adults and contribute to an environment conducive to uric acid stone formation [28]. In some cases, UA is a nidus for calcium salt crystallization, forming mixed stones. These mixed stones require a dual management approach, addressing the UA component through alkalinization and the calcium component by managing hypercalciuria or hypocitraturia [3,6].

Calcium phosphate stones

Calcium phosphate is a common component identified in approximately 85% of urinary stones, with proportions ranging from 0.5% to 99%. Its clinical significance depends on the crystalline phase, location, and overall content within the stone. Recent studies have emphasized the role of carbapatite Randall's plaque as a nidus for calcium oxalate stone formation, where the calcium phosphate content is typically low (<5%). As carbapatite formation is highly pH-dependent, stones rich in carbapatite tend to develop in poorly acidic to alkaline urine, which may result from urinary tract infections (UTIs) or metabolic conditions that chronically elevate urinary pH, with or without hypercalciuria. Additionally, carbapatite associated with brushite or octacalcium phosphate pentahydrate (OCPP) is frequently a marker of hypercalciuria, with OCPP indicating a recent and active lithogenic process [2,3].

The relationship between renal tubular acidosis (RTA) and calcium phosphate stone formation is well-documented and primarily driven by the metabolic disturbances associated with RTA. In RTA, particularly distal RTA (dRTA), the kidney's ability to acidify the urine is impaired due to a defect in hydrogen ion secretion by the distal tubules. This results in persistently alkaline urine, a key factor that promotes calcium phosphate precipitation, primarily in hydroxyapatite or brushite.

The urine's alkalinity reduces the solubility of phosphate, favouring its combination with calcium to form insoluble calcium phosphate crystals. Additionally, the chronic metabolic acidosis seen in RTA leads to bone resorption, increasing calcium and phosphate availability in the bloodstream and their filtration and concentration in the urine. This hypercalciuria, combined with hypocitraturia (a common feature in RTA due to increased citrate reabsorption in the proximal tubule), further exacerbates the risk of stone formation, as citrate normally inhibits calcium crystallization.

Thus, the interplay of urinary alkalinity, hypercalciuria, and hypocitraturia creates an environment conducive to calcium phosphate stone formation in individuals with RTA. Understanding this relationship is crucial for effectively managing and preventing stones in affected patients [29,30]. UTIs are a standard mechanism underlying phosphate stone formation. Stones associated with UTIs often include other calcium phosphate species, such as amorphous carbonated calcium phosphate or whitlockite, alongside carbapatite. The presence of struvite in the stone is a specific indicator of UTI-induced calculi. In cases without struvite, the carbonation rate of carbapatite, as determined by infrared spectroscopy, can serve as an additional diagnostic marker [6,7].

Brushite is a particular form of calcium phosphate stone. Composed of calcium hydrogen phosphate dihydrate, brushite stones are rare stone types associated with metabolic abnormalities. They are notable for their hardness and resistance to fragmentation, making their management more challenging than other stone types. Brushite stones typically form in an alkaline to neutral urinary pH range (6.0-6.5), where the solubility of calcium phosphate decreases, creating favourable conditions for crystallization [3,6]. Brushite stones, composed primarily of calcium phosphate, are known for their high density and hardness, often rendering ESWL less effective. Consequently, PCNL is generally recommended for managing large or complex brushite stones, as it removes these resilient calculi. For smaller brushite stones in the ureter, URS is typically preferred, offering a minimally invasive approach to access and extract or fragment the stones [31]. Medical management focuses on dietary modifications, such as reducing sodium and protein intake to decrease urinary calcium excretion and ensuring adequate hydration to dilute urinary solutes and reduce supersaturation. Potassium citrate supplementation is also recommended to lower urinary calcium phosphate supersaturation and increase urinary citrate levels, reducing the likelihood of stone formation. Additionally, thiazide diuretics are prescribed to address hypercalciuria by enhancing calcium reabsorption in the renal tubules. Brushite stones are prone to high recurrence rates, especially if underlying metabolic abnormalities remain uncorrected [2,7].

Struvite stone

Struvite calculi, also known as infection stones or magnesium ammonium phosphate stones, are urinary stones strongly linked to UTIs caused by urease-producing bacteria. Although less common than calcium-based stones, they are clinically significant due to their rapid growth and potential to form staghorn calculi. Struvite stones develop in the presence of urease-producing bacteria such as *Proteus Mirabilis, Klebsiella Pneumoniae, *and *Pseudomonas Aeruginosa,* which hydrolyze urea into ammonia and carbon dioxide [2,3]. This process increases urinary pH, creating an alkaline environment (pH>7.5), favouring struvite and carbonate apatite precipitation. Struvite crystals often co-precipitate with carbonate apatite in alkaline urine, proliferating large stones and obstructing the renal pelvis and calyces [4,6]. The primary treatment for complex struvite stones, particularly staghorn calculi, is PCNL, which allows direct removal of the stones. URS is adequate for smaller stones, while ESWL may be used for small fragments, though it is less effective for larger or harder stones [32,33].

Effective infection control is critical for preventing recurrence. Long-term antibiotic therapy targeted at eradicating urease-producing bacteria is essential, with culture-specific antibiotics being the most effective. In recurrent cases, long-term suppressive therapy may be necessary. In addition, urinary acidification with agents like L-methionine can help prevent stone formation by reducing the alkaline environment conducive to struvite precipitation [2]. By metabolizing sulfuric acid, L-methionine promotes urinary acidification. Prolonged use of L-methionine can lead to hypercalciuria, which may increase the risk of calcium stone formation. Additionally, systemic acidosis resulting from extended therapy can contribute to bone demineralization. In patients receiving L-methionine for urinary acidification, the target urinary pH should ideally range between 5.8 and 6.2. This range effectively prevents the formation of stones associated with alkaline urine, such as infection-related calculi, while minimizing the risk of excessive acidification that could lead to complications. Despite effective surgical and medical management, struvite stones have a high recurrence rate if the underlying infection is not eradicated [34].

Urinary catheters and JJ stents are significant risk factors for infections that can form struvite stones [35, 36]. These devices provide a surface for bacterial adhesion and biofilm formation, particularly by urease-producing bacteria such as Proteus mirabilis and Klebsiella Pneumoniae. The biofilm creates a microenvironment that facilitates urea hydrolysis, producing ammonia and subsequent alkalinization of the urine (pH>7.5). This process promotes the precipitation of magnesium ammonium phosphate (struvite) and carbonate apatite crystals, increasing the risk of stone formation. Additionally, prolonged catheterization or stent use exacerbates urinary stasis and chronic infections, both of which are critical contributors to the development and recurrence of struvite stones [37].

Cystine stones

Cystine stones, caused by the autosomal recessive condition cystinuria, are a rare form of urinary calculi characterized by excessive cystine excretion due to defective renal tubular reabsorption of dibasic amino acids. Cystine's low solubility in urine, particularly at acidic pH levels below 7.0, leads to supersaturation and the formation of hexagonal crystals that aggregate into stones [6,7]. Management focuses on reducing urinary cystine concentration and increasing its solubility through aggressive hydration (3-4 liters daily) and urinary alkalinization to maintain a pH between 7.0 and 7.5 using agents such as potassium citrate. The primary therapeutic goal is to sustain urinary pH>7.5 to increase cystine solubility and prevent crystallization. This is achieved with alkalinizing agents like potassium citrate or sodium bicarbonate, and home urine pH monitoring is recommended to allow self-adjustment of treatment. Ensuring appropriate hydration, with a minimum intake of >3L/day in adults, is a cornerstone of management [2,3].

Thiol agents, which reduce cystine to cysteine and form more soluble disulfides, are a cornerstone of pharmacologic management. These include tiopronin and penicillamine, among the most used agents for cystine stone management. Tiopronin is often preferred due to its lower toxicity profile than penicillamine, significantly lowering urinary cystine levels. Penicillamine, while effective, is associated with higher rates of adverse effects such as rash, proteinuria, and gastrointestinal disturbances, limiting its long-term use. Acetylcysteine, though historically used, is less commonly employed in modern practice due to the availability of more effective agents. However, it remains a part of the therapeutic armamentarium for patients who may not tolerate other treatments or in settings where other agents are unavailable [38]. Tiopronin is the preferred pharmacological agent for patients with urinary cystine levels > 3.0 mmol/day (720 mg/day) or recurrent stones despite preventive measures. It acts as a reductive substance by breaking the disulfide bonds in cystine, reducing free cystine concentrations. However, potential side effects, including nephrotic syndrome and poor patient compliance, limit its long-term use [39]. ESWL is less effective due to cystine stones' high density and hardness. Cystine stones have a high recurrence rate, with approximately 50% of patients experiencing recurrence within two years if not managed appropriately. However, adherence to hydration, alkalinization, and pharmacological therapies significantly reduces recurrence risk and preserves renal function. Early diagnosis and a comprehensive, lifelong management plan are crucial for minimizing complications, such as chronic kidney disease, in patients with cystinuria [39,40].

Rare kidney stones

In addition to the more common calcium oxalate, calcium phosphate, and uric acid stones, rare kidney stones composition may be associated with unique metabolic, infectious, or drug-related conditions. Drug-induced calculi represent about 1-2% of all renal calculi. Due to their unusual composition and specific pathophysiological mechanisms, these stones may require specialized diagnostic and therapeutic approaches. Certain medications, such as indinavir, can directly contribute to the formation of kidney stones through crystallization in the urinary tract. Formed from the crystallization of the HIV protease inhibitor indinavir, these stones often occur in acidic urine. Triamterene stones are associated with prolonged use of the potassium-sparing diuretic triamterene, and these stones are found in acidic urine [41,42].

## Conclusions

This review highlights the importance of accurate stone composition analysis in managing and preventing urolithiasis, with infrared spectroscopy being a highly reliable method for precisely identifying stone components. Spectroscopy enables tailored treatment strategies and targeted prevention of recurrence, particularly for patients at higher risk. For individuals prone to recurrent stones or belonging to high-risk groups, such as those with metabolic disorders or genetic predispositions, more frequent follow-ups and periodic monitoring are essential. By integrating spectroscopy into standard practice, alongside hydration, dietary modifications, pharmacological interventions, and regular monitoring, clinicians can achieve more effective management and improve long-term outcomes for patients with urinary stones.
